# Delineating inter- and intra-antibody repertoire evolution with AntibodyForests

**DOI:** 10.1093/bioinformatics/btaf560

**Published:** 2025-10-09

**Authors:** Daphne van Ginneken, Valentijn Tromp, Lucas Stalder, Tudor-Stefan Cotet, Sophie Bakker, Anamay Samant, Sai T Reddy, Alexander Yermanos

**Affiliations:** Center for Translational Immunology, University Medical Center Utrecht, Lundlaan 6, 3584 EA Utrecht, The Netherlands; Center for Translational Immunology, University Medical Center Utrecht, Lundlaan 6, 3584 EA Utrecht, The Netherlands; Department of Biosystems Science and Engineering, ETH Zurich, Klingelbergstrasse 48, 4056 Basel, Switzerland; Department of Biosystems Science and Engineering, ETH Zurich, Klingelbergstrasse 48, 4056 Basel, Switzerland; Center for Translational Immunology, University Medical Center Utrecht, Lundlaan 6, 3584 EA Utrecht, The Netherlands; Department of Biosystems Science and Engineering, ETH Zurich, Klingelbergstrasse 48, 4056 Basel, Switzerland; Department of Biosystems Science and Engineering, ETH Zurich, Klingelbergstrasse 48, 4056 Basel, Switzerland; Botnar Institute of Immune Engineering, Klingelbergstrasse 48, 4056 Basel, Switzerland; Center for Translational Immunology, University Medical Center Utrecht, Lundlaan 6, 3584 EA Utrecht, The Netherlands; Department of Biosystems Science and Engineering, ETH Zurich, Klingelbergstrasse 48, 4056 Basel, Switzerland; Botnar Institute of Immune Engineering, Klingelbergstrasse 48, 4056 Basel, Switzerland

## Abstract

**Motivation:**

The rapid advancements in immune repertoire sequencing, powered by single-cell technologies and artificial intelligence, have created unprecedented opportunities to study B cell evolution at a novel scale and resolution. However, fully leveraging these data requires specialized software capable of performing inter- and intra-repertoire analyses to unravel the complex dynamics of B cell repertoire evolution during immune responses.

**Results:**

Here, we present AntibodyForests, software to infer B cell lineages, quantify inter- and intra-antibody repertoire evolution, and analyze somatic hypermutation using protein language models and protein structure.

**Availability and implementation:**

This R package is available on CRAN and Github at https://github.com/alexyermanos/AntibodyForests, a vignette is available at https://cran.case.edu/web/packages/AntibodyForests/vignettes/AntibodyForests_vignette.html.

## 1 Introduction

**Figure 1.**
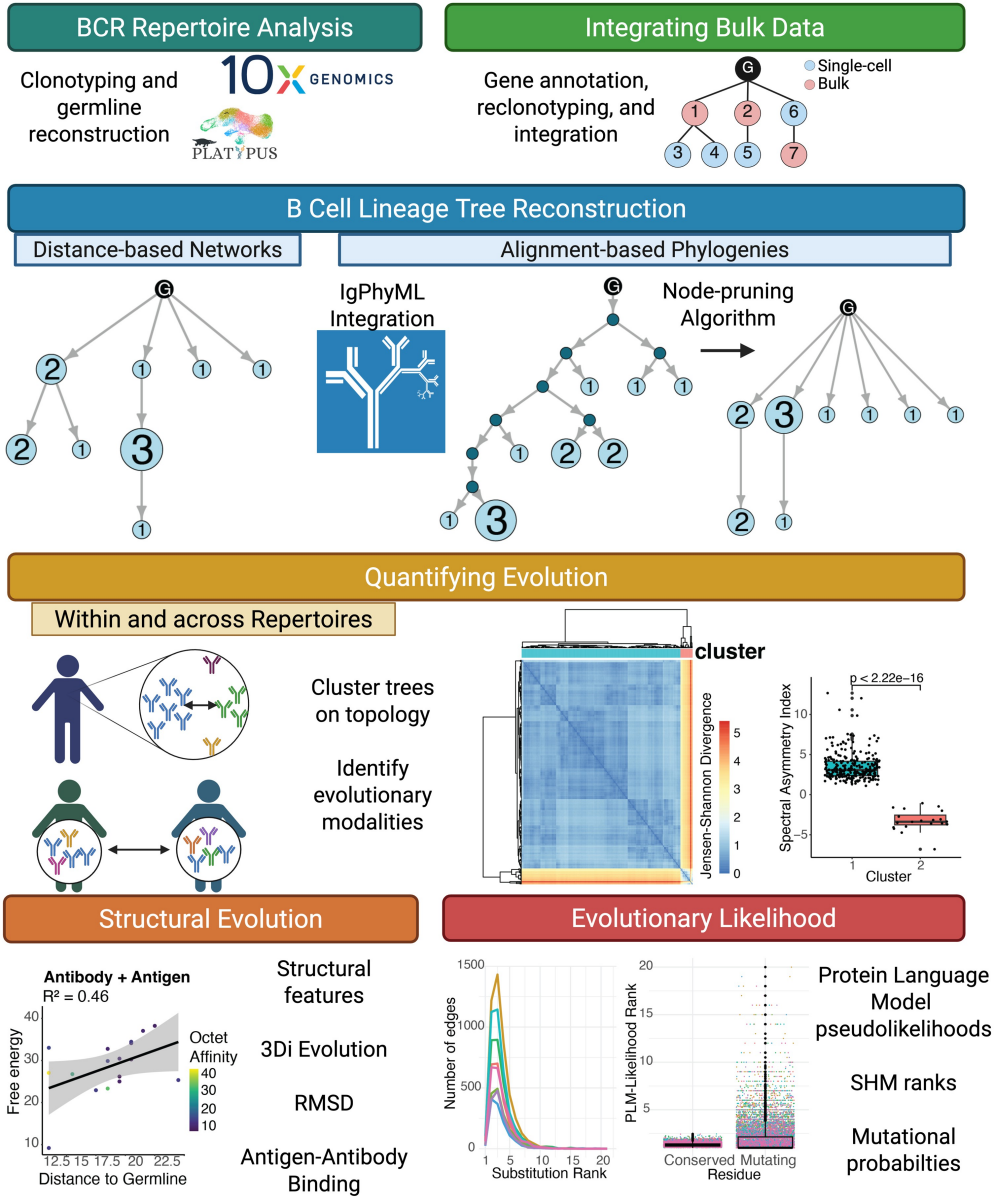
Overview of the AntibodyForests package. This R package is currently composed of a pipeline to reconstruct lineage trees from V(D)J sequencing data preprocessed with the Platypus package and compare trees within and across repertoires. Furthermore, it has modalities to integrate bulk RNA sequencing data, features of protein structure, and evolutionary likelihoods generated with protein language models. Created in BioRender https://BioRender.com/p38k845.

Rapid progress in immune repertoire sequencing and artificial intelligence are advancing the field by providing high-quality datasets at single-cell resolution and pre-trained large protein language models (PLMs). These datasets of paired heavy- and light-chain sequences can additionally include informative labels on cellular phenotype, antigen-binding and specificity, as well as protein structure. Moreover, the integration of bulk RNA sequencing data can significantly enhance the resolution of immune repertoire analyses and reduce undersampling issues common to single-cell experiments. Pre-trained PLMs have demonstrated the ability to understand structural and functional properties from protein sequences and have been used to predict features of antibody function (Ruffolo *et al.* 2021, Leem *et al.* 2022, Olsen *et al.* 2022, Hie *et al.* 2024, van Ginneken *et al.* 2025, Singh *et al.* 2025). The modalities of this data play a critical role in unraveling the evolutionary processes of B cells during immune responses. Numerous analyses and tools focus on inferring and quantifying individual antibody lineages (Barak *et al.* 2008, Gupta *et al.* 2015, Schramm *et al.* 2016, Hoehn *et al.* 2017, DeWitt *et al.* 2018, Lindeman *et al.* 2018, Hoehn *et al.* 2022, Jensen *et al.* 2024), but there is a lack of software dedicated to studying antibody sequence and structural evolution at the repertoire level. In response to this gap, we introduce AntibodyForests, a comprehensive software designed to thoroughly investigate and quantify the inter- and intra-antibody repertoire evolution (Fig. 1). AntibodyForests integrates pipelines for analyzing sequence data, associated metadata, phylogenetic tree structures, protein conformations, and PLM features. It introduces novel capabilities beyond existing tools for antibody repertoire phylogenetic analysis, while maintaining comparable computational efficiency (Barak *et al.* 2008, Schramm *et al.* 2016, DeWitt *et al.* 2018, Lindeman *et al.* 2018, Hoehn *et al.* 2022) (Tables 1 and 2, available as supplementary data at *Bioinformatics* online).

## 2 Usage and application

### 2.1 Tree reconstruction

AntibodyForests infers B cell mutational networks for reconstructed clonal lineages (clonotypes) (Fig. 1, available as supplementary data at *Bioinformatics* online). These clonotypes are defined as B cells arising from the same V(D)J recombination event that have undergone somatic hypermutation (SHM) relative to an unmutated reference germline. In AntibodyForests, each clonal lineage is represented as a graph in which the nodes refer to unique antibody sequences, and the edges separating the nodes dictate the clonal relationship between variants. These nodes can be full-length V(D)J sequences, or only certain gene segments or complementarity-determining regions (CDR) from the heavy and/or light chain.

AntibodyForests contains several tree construction algorithms based on either distance matrices or multiple sequence alignments and incorporates minimum spanning tree algorithms and phylogenetic methods. For the distance-based algorithm, AntibodyForests starts by creating a distance matrix based on a user-supplied sequence distance metric. Next, AntibodyForests can create a germline-rooted minimum spanning tree or neighbor-joining tree. Alternatively, the default algorithm of AntibodyForests starts with the germline node and iteratively adds nodes with the smallest distance, comparable with previously developed algorithms (Barak *et al.* 2008). In the case of a tie during the default network construction, AntibodyForests has several options that allow the user to select whether breadth or depth (i.e. whether new sequences are added to the node with the most or least descendants), mutational load (whether they are added to the node closest or furthest from the germline), clonal expansion (whether they are added to the node with the highest or lowest cell count), or random addition dictates network topology. For the phylogenetic algorithms, AntibodyForests starts by creating a multiple sequence alignment. Next, AntibodyForests can construct a maximum parsimony tree, minimizing the number of mutations, or a maximum likelihood tree based on various evolutionary substitution models. Alternatively, users can supply previously computed phylogenetic trees in Newick format, thereby enabling the integration of different mutational models, such as those developed specifically for antibodies (Yaari *et al.* 2013, Mirsky *et al.* 2015, Hoehn *et al.* 2017). To maximize flexibility for the user, AntibodyForests is additionally compatible with the output format of the commonly used IgPhyML tool (Hoehn *et al.* 2019). AntibodyForests can further interrogate and visualize network robustness by comparing tree topology across different tree construction methods using metrics such as the generalized branch length distance (GBLD) (Farnia and Tahiri 2024) (Fig. 4, available as supplementary data at *Bioinformatics* online). Although the package is designed for single-cell immune repertoire data, the pipeline can also be used with bulk repertoire sequences and even has a function to integrate the two data modalities (Fig. 3, available as supplementary data at *Bioinformatics* online).

Our framework diverges from the traditional B cell phylogenetic tree structure by enabling recovered sequences to serve as either internal or terminal nodes, in addition to allowing for multifurcation events. Some phylogenetic methods generate internal nodes serving as the most recent common ancestors (MRCA). In antibody repertoires, these MRCAs can be recovered in the sequencing data (Barak *et al.* 2008, Davidsen and Matsen 2018, Hoehn and Kleinstein 2024), resulting in nodes with zero branch length to the internal parent node, or they can represent B cells that are not part of the repertoire at the time of sampling (Fig. 2, available as supplementary data at *Bioinformatics* online). To address this, AntibodyForests offers flexibility of handling internal nodes, allowing users to tailor tree representations to either lineage networks of sampled sequences or phylogenetic trees with unsampled intermediate cells. To transform traditional bifurcating phylogenetic trees into multifurcating lineage networks, AntibodyForests includes various algorithms to remove these inferred internal nodes. For example, users can choose to remove only internal nodes with zero branch length to a terminal node, which preserves mutational ordering. Additionally, internal nodes can be replaced by their closest descendant or directly linked to an upstream parental node, favoring either depth or breadth in tree topology. Removing internal nodes potentially impacts the evolutionary trajectory and thereby influences downstream analyses and interpretations; however, how different methods impact tree topology can be quantified using functions within AntibodyForests. When comparing different trees and repertoires to each other, it is important to use the same method for internal node removal.

Upon completion of the network, the color of each node can correspond to the fraction of cells with a given isotype, transcriptional cluster (if such information is available), or a custom BCR feature (Fig. 2, available as supplementary data at *Bioinformatics* online). To highlight receptor sequences that are identical across multiple B cells, the node size can be scaled to match the relative clonal expansion, while the label can be adjusted to depict the variant frequency. To understand the evolutionary distance separating two nodes, it is possible to include edge labels that correspond to the distance separating nodes.

### 2.2 Quantifying intra- and inter-antibody repertoire evolution

A quantitative interpretation of antibody lineage trees is crucial to identify different patterns of clonal selection and expansion that drive B cell evolution within and across repertoires. A repertoire-wide analysis of these tree topologies can identify features of SHM and could identify potentially interesting therapeutically relevant antibodies or vaccine targets. For example, Wu *et al.* discovered a broadly neutralizing antibody against HIV-1 by identifying progenitor sequences preceding viral neutralization capabilities using phylogenetics (Wu *et al.* 2011).

Phylogenetic methods have been extensively used to investigate single B cell clonal lineages (Wu *et al.* 2011, Liao *et al.* 2013, Cizmeci *et al.* 2021, Kim *et al.* 2022, Neumeier *et al.* 2022, Jensen *et al.* 2024). However, the evolution of the repertoire as a system has not been fully explored using the collection of lineage trees. AntibodyForests includes two modules explicitly dedicated to comparing evolution within and across repertoires, in addition to comparing topologies consisting of identical sequences (Fig. 4, available as supplementary data at *Bioinformatics* online). Tree topology metrics can be either calculated and stored within the AntibodyForests object or into a separate matrix that can be supplied for downstream clustering and visualization. We have included various metrics to describe tree topologies, including general graph theory metrics and more specialized metrics. For example, we included the Sackin index for tree imbalance, where a high index represents longer branches and more nodes arising from a specific descendant and thereby suggests the presence of selective pressure (Sackin 1972). Users can also compute various properties of the Laplacian spectral density of the lineage trees. These properties characterize specific evolutionary patterns such as species richness (principal eigenvalue), deep or shallow branching events (asymmetry), and tree imbalance (peakedness) (Lewitus and Morlon 2016). Together, AntibodyForests’ metrics can be used to project topology features using dimensionality reduction techniques and clustering, thereby identifying topologically similar networks. Clusters of trees can be related to features specific to single-cell immune repertoire sequencing, such as transcriptional phenotype, isotype, or expansion, as well as other custom metadata features provided by the user.

To illustrate the suitability of AntibodyForests to comprehensively reconstruct and analyze whole BCR repertoires and study patterns of SHM upon immune activation, we included example analyses in the Supplementary Material using single-cell BCR data collected from the blood and lymph nodes of individuals post SARS-CoV-2 vaccination (Kim *et al.* 2022). Using this dataset, we demonstrate example analyses and output plots that arise directly from functions internal to AntibodyForests while also confirming results present in the original paper (Fig. 5A–E, available as supplementary data at *Bioinformatics* online). This includes comparing tree topologies both within and across repertoires using the aforementioned metrics, and we further provide examples on how users can seamlessly relate repertoire features such as V-gene usage and isotype to evolutionary features.

### 2.3 Relating protein language model likelihoods to B cell evolution

PLMs have demonstrated success in understanding features of sequence, structure, and function of natural proteins (general PLMs) (Jumper *et al.* 2021, Rives *et al.* 2021, Elnaggar *et al.* 2022, Lin *et al.* 2023). Following this success, additional PLMs trained specifically on antibody sequences were able to learn B cell specific features such as germline usage and SHM (antibody-specific PLMs) (Ruffolo *et al.* 2021, Leem *et al.* 2022, Olsen *et al.* 2022, 2024, Singh *et al.* 2025, van Ginneken *et al.* 2025. The learning objective of PLMs allows for the computation of a likelihood score for each residue in the input sequence. These scores can be used to calculate a so-called pseudolikelihood for the entire sequence. PLM-based pseudolikelihoods have been leveraged to capture global evolutionary trajectories (Hie *et al.* 2022). Furthermore, general PLMs have been demonstrated to improve antibody affinity through in silico mutagenesis by mutating for increasing evolutionary likelihood (Hie *et al.* 2024). Given the relevance of PLMs in studying and engineering antibodies, we included PLM-guided metrics within AntibodyForests to describe B cell evolution at the level of an entire repertoire and a single lineage. Both per-residue likelihoods and per-sequence pseudolikelihoods can be incorporated into AntibodyForests to evaluate correlations between tree topology and PLM-based likelihoods of mutating and conserved residues along the edges of the trees and compare within and across repertoires (Fig. 6A, available as supplementary data at *Bioinformatics* online). We have provided examples of how AntibodyForests can be used to compare PLM-based likelihoods both within and across repertoires and have further highlighted the types of plots that can be directly produced with functions internal to AntibodyForests (Fig. 6B–E, available as supplementary data at *Bioinformatics* online). Users are able to compare how PLM-based likelihoods change as a function of B cell evolution, which can provide insight into which residues may be more likely to evolve and which amino acids are tolerated during SHM. The computational pipeline present in AntibodyForests can help users perform PLM-guided antibody engineering (Hie *et al.* 2024) and further be used to study selection signals of antibody repertoires (van Ginneken *et al.* 2025. Importantly, we have ensured that AntibodyForests is compatible with many types of PLMs, including both general and antibody-specific PLMs. Users can avoid potential biases that may be present in antibody databases, such as the high proportion of germline-like antibodies, by leveraging PLMs trained on diverse types of protein sequences that also include filters based on homology thresholds [e.g. ESM family of PLMs (Rives *et al.* 2021, Lin *et al.* 2023)], by fine-tuning custom PLMs, or by using PLMs explicitly trained to avoid germline biases such as Ablang2 (Olsen *et al.* 2024).

### 2.4 Structural evolution

Recent computational efforts have massively accelerated the ability to predict protein structures from sequence alone (Baek *et al.* 2021, Jumper *et al.* 2021, Lin *et al.* 2023, Ruffolo *et al.* 2023). Although SHM acts on the sequence directly, the structure of the antibody determines binding affinity with antigens. We therefore included a structure-specific module in AntibodyForests to integrate antibody structural evolution (Fig. 7A, available as supplementary data at *Bioinformatics* online). This allows users to quantify structural features as a function of sequence distance from the germline. Examples of these features are: (i) structure similarity [root-mean-square deviation (RMSD)], (ii) biophysical properties [hydrophobicity, charge, 3Di alphabet (van Kempen *et al.* 2024), free energy, and pKa (Olsson *et al.* 2011)], and (iii) global confidence of the prediction [predicted Local Distance Difference Test (pLDDT)]. Furthermore, we have added the option to include antibody-antigen complexes and calculate how properties at the binding interface change with SHM. In addition to RMSD to the germline, AntibodyForests can calculate a pairwise RMSD between the structures connected with edges in the lineage trees and calculate their correlation with the number of mutations. To illustrate a use case of the AntibodyForests structural functions, we highlight how AntibodyForests can integrate structures from Alphafold3 of antibody-antigen complexes to quantify structural evolution within individual lineages (Fig. 7, available as supplementary data at *Bioinformatics* online). This functionality enables detailed analyses of how SHM, in response to immune stimuli such as vaccination, may drive structural divergence and functional maturation. Although structural modeling methods still struggle with the complex and variable nature of antibody CDR loops and individual mutations on structural predictions (Abanades *et al.* 2022, Ruffolo *et al.* 2022, 2023), the framework included in AntibodyForests will remain relevant as deep learning algorithms continue to improve.

## 3 Concluding remarks

Taken together, AntibodyForests can both infer and visualize individual clonal lineages from single-cell V(D)J sequencing data and incorporate data such as bulk V(D)J sequences and transcriptional phenotypes. AntibodyForests can incorporate trees constructed with existing tools, and transform its bifurcating phylogenetic output to a more accurate multifurcating representation of the inferred antibody lineages. The package hosts various algorithms to compare evolution within and across repertoires using graph theory, protein language models, and protein structure. With the ongoing increase in immune repertoire sequencing data and the continuous improvement of deep learning strategies, we believe AntibodyForests is an invaluable toolbox to analyze B cell evolution and selection at both the clonal and repertoire levels. 

## Supplementary Material

btaf560_Supplementary_Data

## Data Availability

The accession numbers and publications for the sequencing data used in this manuscript and the AntibodyForests code are located in the Supplementary Data.
