# Supplementary material for: Delineating inter- and intra-antibody repertoire evolution with AntibodyForests
Source: Bioinformatics. 2025 Oct 9;41(10):btaf560. doi: 10.1093/bioinformatics/btaf560 (PMC12548040; doi:10.1093/bioinformatics/btaf560)
Supplement: btaf560_Supplementary_Data [file btaf560_supplementary_data.pdf]

Delineating inter- and intra-antibody repertoire evolution with AntibodyForests

Daphne van Ginneken[1], Valentijn Tromp[1], Lucas Stalder[2], Tudor-Stefan Cotet[2], Sophie Bakker[1], Anamay Samant[2], Sai T. Reddy[2,3], Alexander Yermanos[1,2,3,*]

[1]Center for Translational Immunology, University Medical Center Utrecht, Lundlaan 6, Utrecht 3584EA, The Netherlands.
[2]Department of Biosystems Science and Engineering, ETH Zurich, Basel, Switzerland.
[3]Botnar Institute of Immune Engineering, Basel, Switzerland
*Corresponding author. Email: a.d.yermanos@umcutrecht.nl

Supplementary data

	AntibodyForests	Dowser (1)	IgTree (2)	SONAR (3)	GCtree (4)	BraCer (5)
**Lineage reconstruction algorithms**						
Distance-based networks	+	-	+	-	-	+
Maximum Parsimony	+	+	-	-	+	-
Maximum Likelihood	+	+	-	+	-	+
B-cell specific substitution model	+	+	-	+	+	-
Recovered sequences as internal nodes	+	-	+	-	+	+
Reconstruct intermediate sequences	-	+	-	+	-	-
Integrate single-cell and bulk sequences	+	-	-	-	-	-
Create lineage tree plots	+	+	-	+	+	+
**Quantifying sequence evolution**						
Topology metrics of trees	+	-	-	-	-	-
Metadata distribution within trees	+	+	-	+	-	-
Compare trees based on topology	+	-	-	-	-	-
Compare (groups of) repertoires based on topology	+	-	-	-	-	-
Cluster trees on topology	+	-	-	-	-	-
Analyze longitudinal samples	+	+	-	+	-	-
Analyze mutations along the edges of the trees	+	-	+	-	-	-
**Quantifying functional evolution**						
Integrate protein structure	+	-	-	-	-	-
Estimated antibody-antigen binding	+	-	-	-	-	-
Protein Language Model likelihoods	+	-	-	-	-	-
**Accessibility**						
Open-source implementation	+	+	-	+	+	+
Vignette available	+	+	-	+	+	+

Table S1. Comparison of AntibodyForests to other tools for the analysis of B cell lineages. AntibodyForests advances other tools with regard to the downstream analysis of the lineage trees.

	**AntibodyForests**	**Dowser** (1)
**Preprocessing***		
CPU time (seconds)	**667.71**	707.31
RAM usage (megabytes)	**861.2**	1,181.6
Wall-clock time (seconds)	708.89	**169.55**
**Maximum Likelihood Tree**		
CPU time (seconds)	508.95	**34.49**
RAM usage (megabytes)	**738.8**	914.6
Wall-clock time (seconds)	514.86	**35.29**
**Maximum Parsimony Tree**		
CPU time (seconds)	**33.47**	49.31
RAM usage (megabytes)	**646.3**	925.3
Wall-clock time (seconds)	**34.04**	50.93
**IgPhyML Tree**		
CPU time (seconds)	587.75	**1.86**
RAM usage (megabytes)	1,076.4	**24.37**
Wall-clock time (seconds)	**7,730.09 (2h8m)**	16,111.44 (4h28m)
**Distance-based Network**		
CPU time (seconds)	**11.54**	*Not available*
RAM usage (megabytes)	**133.2**	*Not available*
Wall-clock time (seconds)	**12.14**	*Not available*

Table S2. Benchmarking AntibodyForest and Dowser (1)**.** Benchmarking runtime and RAM usage of preprocessing CellRanger output and constructing lineage trees for the heavy chain nucleotide sequences of sample SRR17729692 from the Kim et al. dataset (6). *Preprocessing for AntibodyForests uses the Platypus (7) software and Dowser uses Change-O and SHazaM. Preprocessing includes grouping the BCR transcripts into clonotypes, assigning a germline, and preparing a dataframe ready for lineage tree reconstruction. Code available at https://github.com/dvginneken/BenchmarkingAntibodyForests

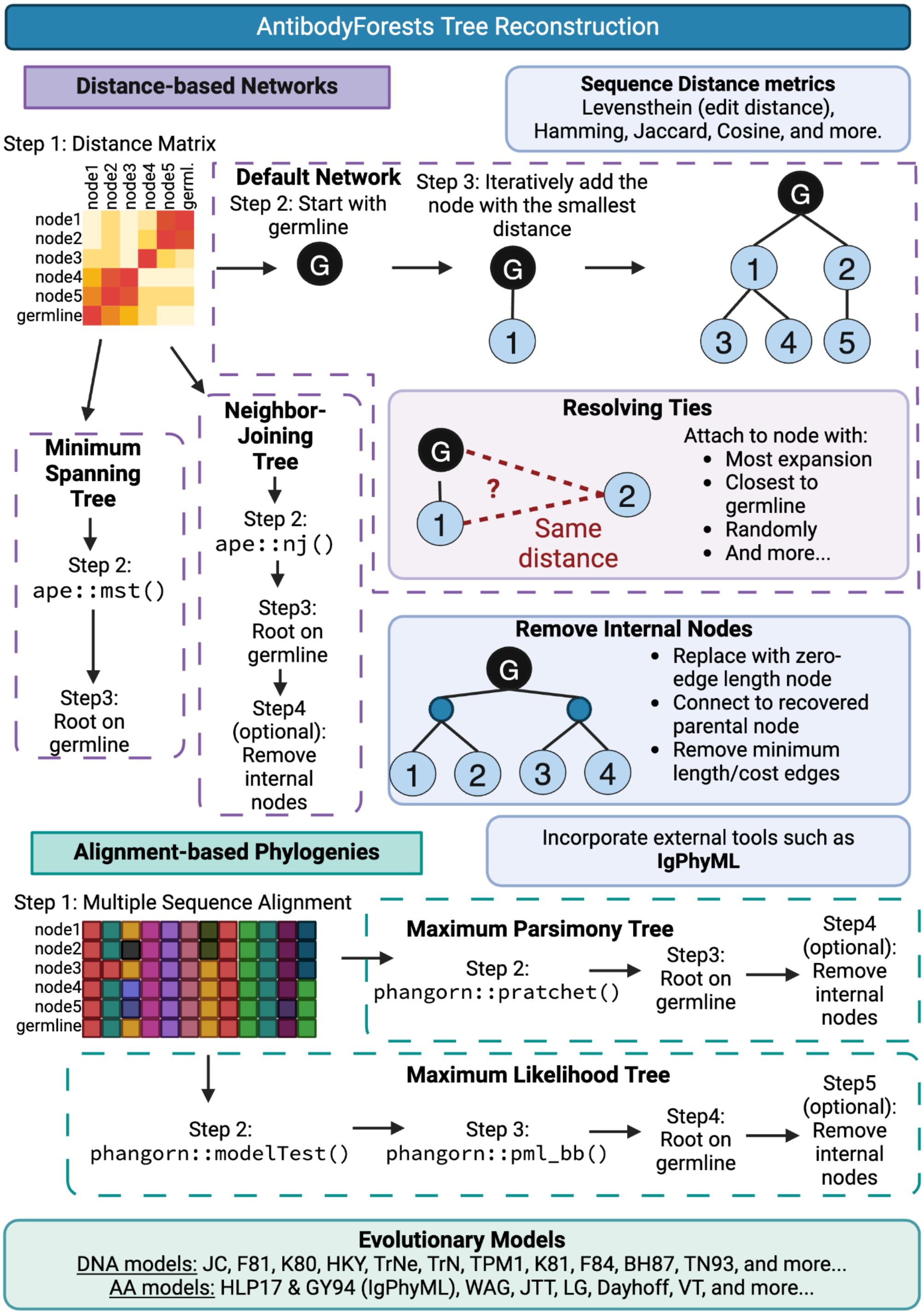

Figure S1. Overview of tree reconstruction algorithms in AntibodyForests. AntibodyForests contains five methods for lineage tree reconstruction and can integrate output from external tools such as IgPhyML (8). AntibodyForests works with various metrics for sequence distance and a wide range of evolutionary substitution models. Additional algorithms

are available to resolve ties and remove internal nodes during network construction. Created in BioRender https://BioRender.com/r71h810.

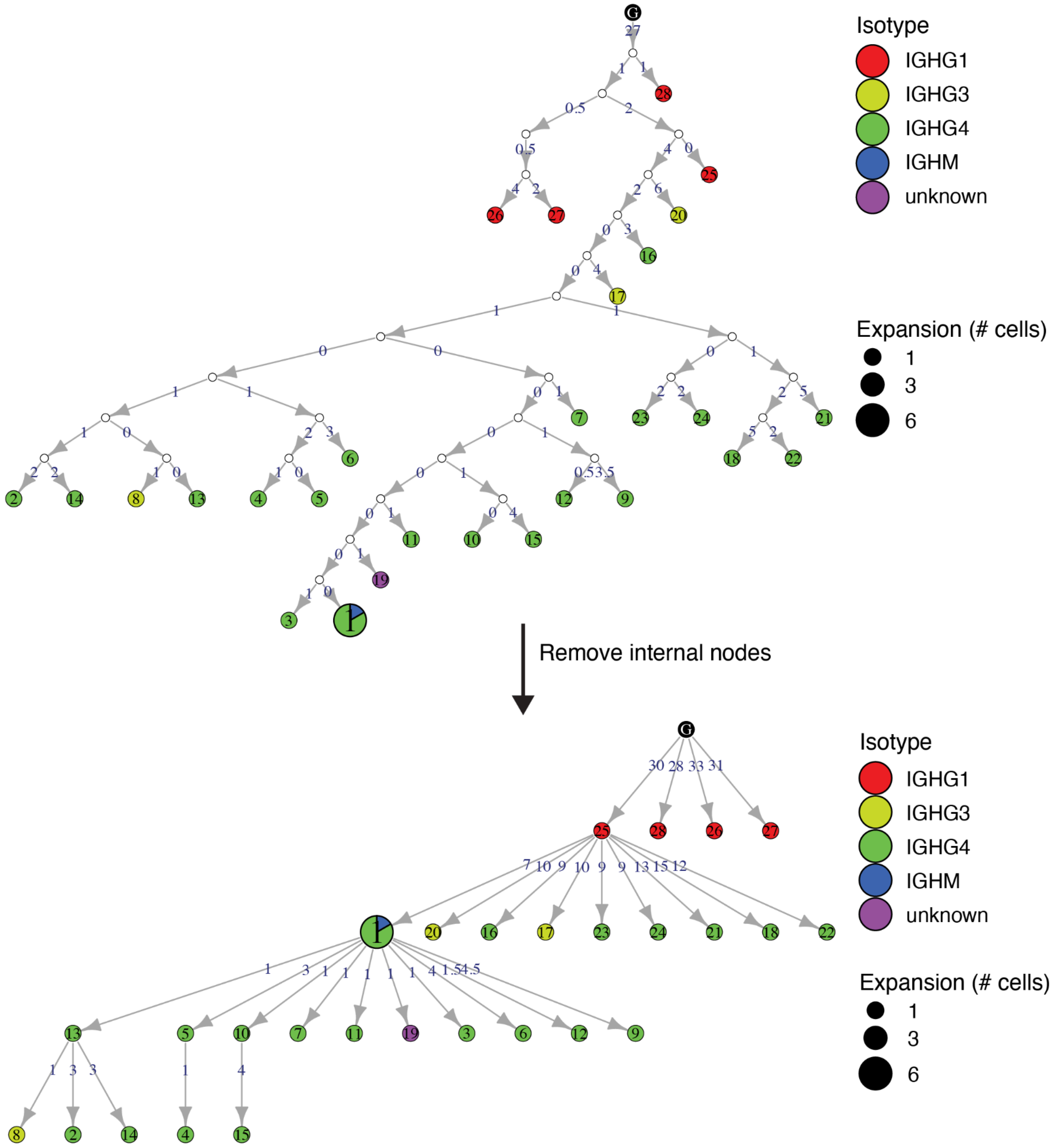

Figure S2. Examples of AntibodyForests trees. An example AntibodyForests object was generated using the maximum parsimony algorithm in the Af_build function. The top tree is before internal node pruning, the bottom tree is after node pruning. The black node on top of the network refers to the germline as indicated by 10x Genomics, the white nodes (top tree) are internal nodes, and the colored nodes are recovered sequences. Node colors correspond to the isotypes. If multiple isotypes are presented within a node, the node is represented as a pie chart. Node size corresponds to the number of cells with that unique heavy+light chain combination. Node labels can be matched to additional information within the AntibodyForests

object. Edge labels correspond to the edit distance between the sequence of the attached nodes. Single-cell BCR data for this analysis was derived from Kim et al. (6)

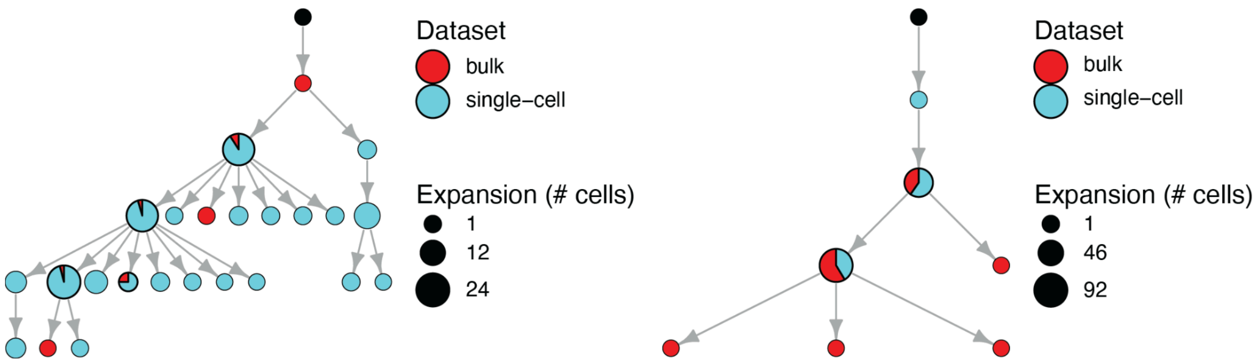

Figure S3. Example bulk RNA integration. An example AntibodyForests object was generated using both single-cell and bulk heavy chain BCR sequencing using the default parameter settings in the VDJ_integrate_bulk and Af_build functions. The black nodes correspond to the reconstructed germlines. These two trees serve as examples to show the identical sequences between the bulk and single-cell datasets (pie chart nodes) and the integration of unique bulk sequences into the single-cell based clonotypes (red nodes). Single-cell and bulk BCR data for this analysis were derived from Neumeier et al. (9).

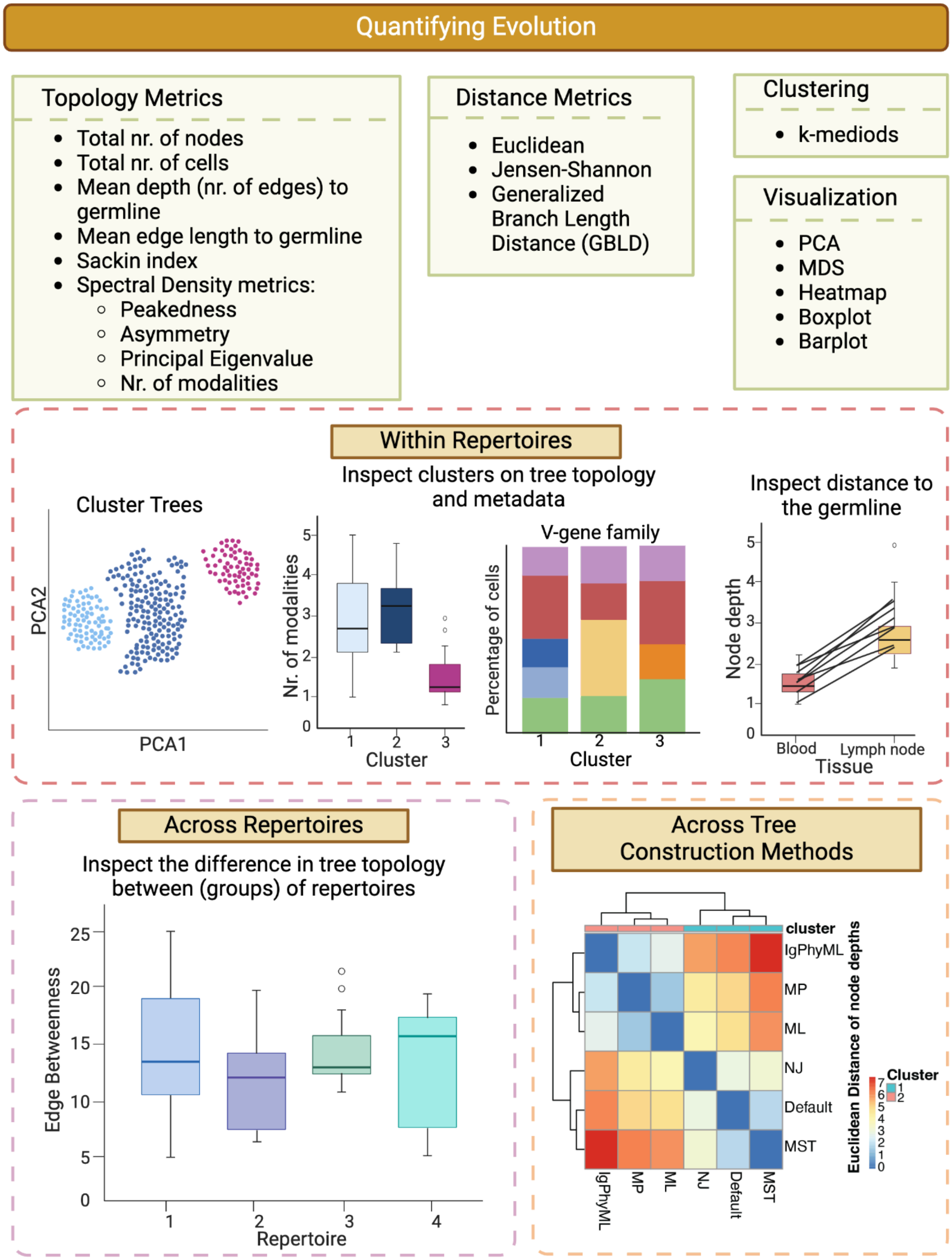

Figure S4. Overview of methods to quantify evolution. AntibodyForests contains metrics to compare within and across repertoires or the same set of trees with different methods. Trees can be clustered based on various distance metrics and visualized in for example dimensionality reduction plots such as a PCA plot. AntibodyForests contains functions to investigate the difference in topology metrics (such as the nr. of spectral density modalities) or metadata of the lineage tree nodes (such as v-gene family) between the clusters. Additionally, distance to the

germline (e.g. node depth) can be compared between metadata features of the nodes. AntibodyForests can additionally compare tree topology metrics across repertoires, such as the edge betweenness (the number of shortest paths between two nodes that cross this edge). To compare different tree reconstruction algorithms, AntibodyForests can be used to create heatmaps of distance metrics between the trees, such as the Euclidean distance of node depths. A more detailed explanation of all metrics can be found in the vignette (https://cran.case.edu/web/packages/AntibodyForests/vignettes/AntibodyForests_vignette.html). Created in BioRender https://BioRender.com/i95h582.

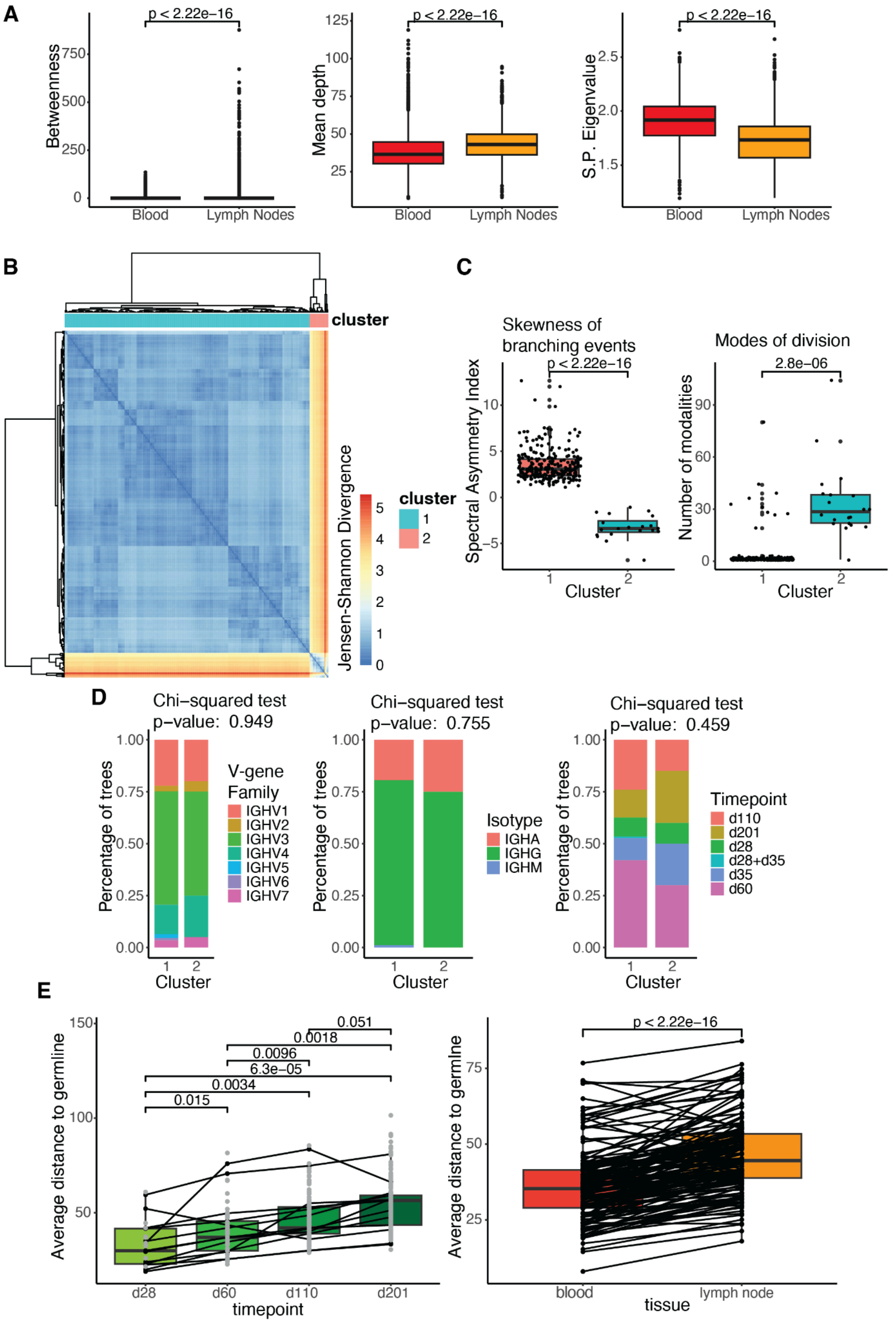
A
Betweenness
p < 2.22e−16
Blood
Lymph Nodes
Mean depth
S.P. Eigenvalue
B
cluster
Jensen-Shannon Divergence
C
Skewness of branching events
Spectral Asymmetry Index
Cluster
Modes of division
2.8e−06
Number of modalities
D
Chi−squared test
p−value: 0.949
Percentage of trees
V-gene Family
IGHV1
IGHV2
IGHV3
IGHV4
IGHV5
IGHV6
IGHV7
p−value: 0.755
Isotype
IGHA
IGHG
IGHM
p−value: 0.459
Timepoint
d110
d201
d28
d28+d35
d35
d60
E
Average distance to germline
timepoint
d28
d60
d110
d201
0.051
0.0018
0.0096
6.3e−05
0.0034
0.015
Average distance to germlne
tissue
blood
lymph node

Figure S5. Example of how AntibodyForests can quantify antibody repertoire evolution of single-cell BCR data collected from the blood and lymph nodes of individuals post SARS-CoV-2 vaccination. B cell lineage trees were constructed with the default algorithm using the heavy+light chains using the Af_build function. A) Comparison of tree topology metrics (node betweenness, average edge length to the germline, and spectral principal eigenvalue) between BCR repertoires in the blood and lymph nodes using the Af_compare_across_repertoire function. This across-repertoire comparison revealed the blood repertoire to have more diversity, while the deeper trees in the lymph node repertoire suggest more sequential evolution. B) The B cells from all tissues and time points for each individual were clonotyped together and the trees were clustered based on the Laplacian spectrum. This heatmap shows the Jensen-Shannon divergence in Laplacian spectral density between trees using the Af_compare_within_repertoires function. C) Spectral density metrics of trees in both clusters using the Af_cluster_metrics function. This within-repertoire comparison demonstrated a subset of trees displaying deep branching events, suggesting that cells with a small degree of SHM were recovered. This same subset was characterized by multiple topology modalities, indicating various diversification events. D) Proportion of trees in each cluster and their predominant labels (V-gene family, isotype, and timepoint of sampling) using the Af_cluster_node_features function. E) Average distance (sum of edge lengths) from the nodes of each group to the germline using the Af_distance_boxplot function. Grey dots represent trees not containing all groups. This analysis revealed that SHM increased with the time after vaccination and that blood-derived BCRs were located closer to the germline than those from lymph nodes. Single-cell BCR data for this analysis was derived from Kim et al. (6)

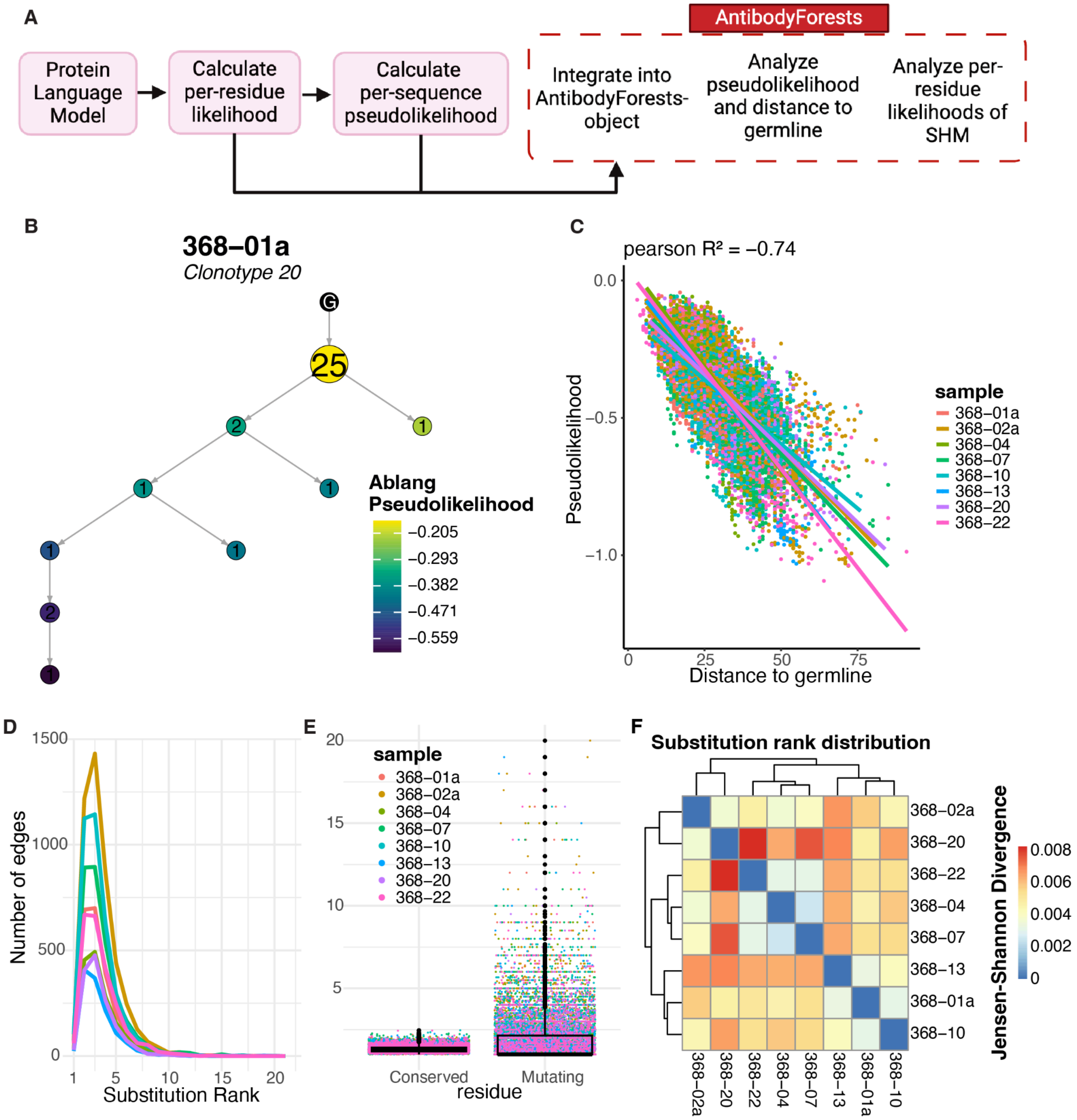

Figure S6. Example of how AntibodyForests can integrate B cell evolution with protein language model (PLM) likelihoods. A) Overview of the PLM workflow integrated with AntibodyForests. After calculating the PLM-based likelihoods of the BCR sequences, AntibodyForests can be employed for integration and downstream analysis. B) An example tree colored on the heavy chain pseudolikelihood using the PLM Ablang (10), which was integrated into the AntibodyForests object using the Af_add_node_feature function. C) The correlation between the pseudolikelihood and the Levenshtein distance to the germline using the Af_distance_scatterplot function. D) The per-residue likelihood rank of the substitution along the edges of the trees using the Af_PLM_dataframe and Af_plot_PLM functions E) The average per-residue likelihood rank of the mutating and conserved residues along the edges of the trees

using the Af_plot_PLM_mut_vs_cons function. F) The Jensen-Shannon divergence of the substitution rank distributions between samples using the Af_compare_PLM function. Single-cell BCR data for this analysis was derived from Kim et al. (6)

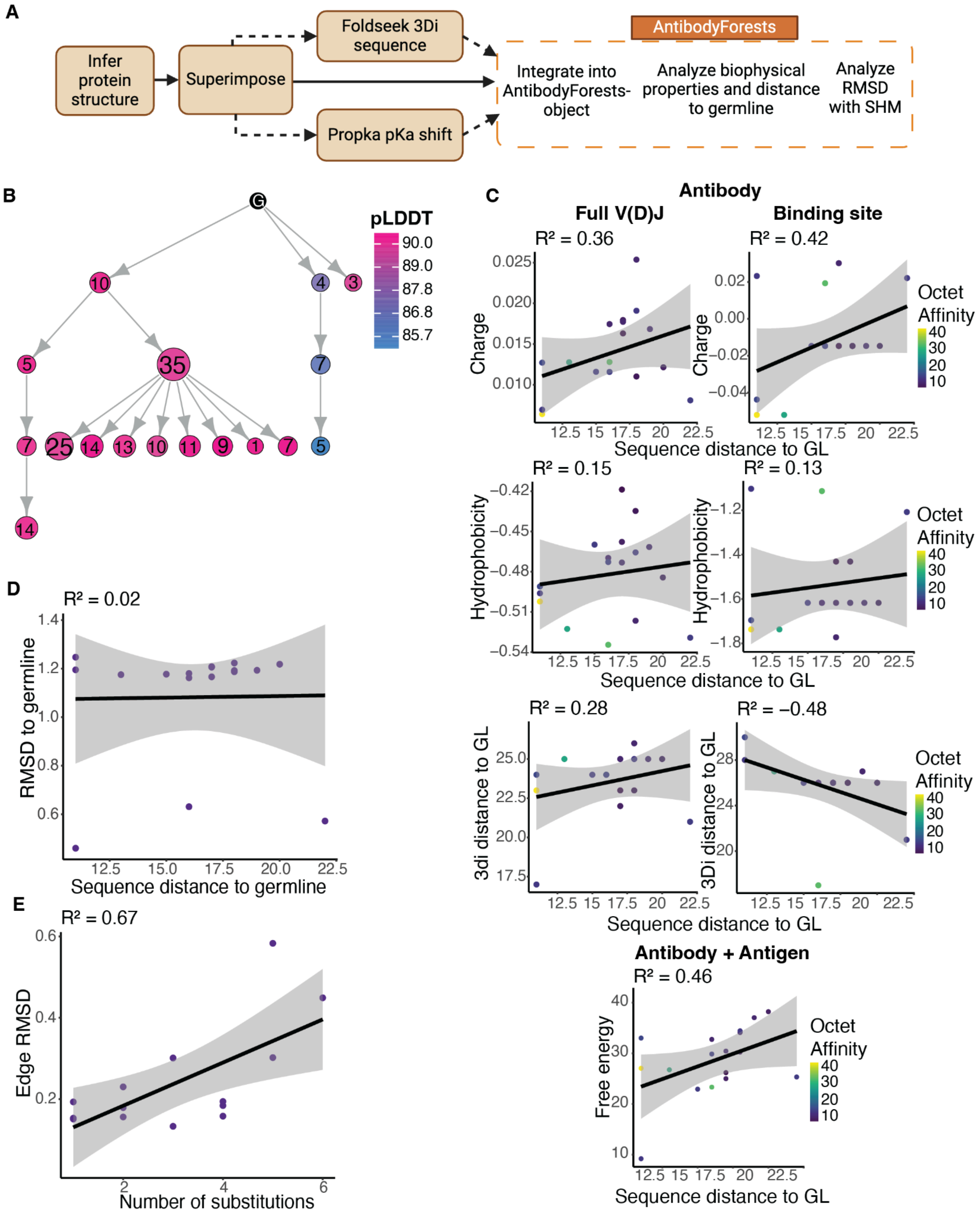

Figure S7. Example of how AntibodyForests can integrate protein structure evolution with sequence evolution. A) Overview of the antibody structure workflow within AntibodyForests. After inferring the 3D structure of the antibody-antigen complexes AntibodyForests can be employed for integration and downstream analysis. B) An example tree colored on the average pLDDT score provided by AlphaFold 3 (11). Lower confidence scores from AlphaFold3 were observed for a specific branch in the lineage tree, suggesting more intrinsically disordered regions. C) The correlation between biophysical properties and the Levenshtein distance to the germline for the full length V(D)J sequence (left) and for the antibody-antigen binding residues (right), and the full antibody-antigen complex (bottom). These properties were calculated with the VDJ_3d_properties function and integrated into the AntibodyForests object with the function Af_add_node_feature. D) The correlation between the root-mean-square deviation (RMSD) from the germline and the Levenshtein distance to the germline for the full length antibody generated with the VDJ_3d_properties function. E) The correlation between the RSMD and the number of substitutions over each edge in the tree generated with the Af_edge_RMSD function. Single-cell BCR and binding affinity data for this analysis were derived from Neumeier et al. (9)**.**

Online methods

Data analysis: For **Figures S2**, **S5,** and **S6**, the raw BCR reads from all samples generated by Kim et al. (6) were processed with 10X Genomics' Cell Ranger and the VDJ_build function of the Platypus package (version 3.6.0) in R (v4.4.0) (7). We applied the following parameters to the VDJ_build() function: trim.germlines = T, remove.divergent.cells = T, complete.cells.only = T. These parameters trim the germline to the V(D)J region, remove cells with more than 1 V(D)J transcript, and remove cells missing either a heavy or light chain transcript. Next, all samples per donor (retrieved at different time points after vaccination and from both the blood and lymph nodes), were reclonotyped using the Platypus function VDJ_clonotype_v3_w_enclone. We applied the following parameters to the VDJ_clonotype_v3_w_enclone() function: clone.strategy = "enclone" and global.clonotype = T. Under these parameters, the function uses 10X Genomics' Enclone tool to group cells from all samples by V- and J-gene and CDR3 length, and shared SHM with a CDR3 nucleotide identity of at least 85%. For **Figure S5A** we only reclonotyped the time points and kept the tissues as separate clonotypes. This reclonotyped VDJ-dataframe obtained with Platypus is then supplied to AntibodyForests.

For **Figure S2**, we subset the VDJ-dataframe to only include donor 368-10. We run the function Af_build to construct lineage trees for each clonotype with the following parameters: sequence.columns = c("VDJ_sequence_aa_trimmed","VJ_sequence_aa_trimmed"), germline.columns = c("VDJ_germline_aa_trimmed","VJ_germline_aa_trimmed"), and construction.method = "phylo.tree.mp". With these parameters, nodes in the trees represent both the heavy and light chain sequence, and the maximum parsimony method is used to construct the trees, by default the Levensthein distance is used as the distance metric. To create the figures, we use the function Af_plot_tree with the following parameters: sample = "368-10", clonotype = "clonotype40", label.by = "name", edge.label = "original", show.inner.nodes = F (for subfigure A) and show.inner.nodes = T (for subfigure B). With these parameters, the lineage tree of clonotype 40 is plotted, the nodes are labeled by their assigned

node names from the AntibodyForests-object, the edges are labeled by Levensthein distance between the respective nodes, and the nodes are by default colored on isotype and sized by expansion.

For **Figure S5**, we run the function Af_build on all samples with the following parameters: sequence.columns = c("VDJ_sequence_aa_trimmed","VJ_sequence_aa_trimmed"), germline.columns = c("VDJ_germline_aa_trimmed","VJ_germline_aa_trimmed"), and construction.method = "phylo.network.default". For the across-repertoire comparison we run the function Af_compare_across_repertoire with the parameters: metrics = c("spectral.density", "mean.edge.length", "betweenness"), plot = “boxplot”, significance = T. For clustering of the trees, we supply the AntibodyForests-object to the function Af_compare_within_repertoires with the following parameters: min.nodes = 20, distance.method = "jensen-shannon", clustering.method = "mediods", visualization.methods = "heatmap". With these parameters, we only consider trees with at least 20 nodes for topology analysis. We perform a k-mediods clustering on Jensen-Shannon divergence and create a heatmap. To inspect the difference in topology metrics between the clusters, we use the function Af_cluster_metrics with the parameters: clusters = output$clustering (output from Af_compare_within_repertoires), metrics = "spectral.density", min.nodes = 20, and significance = T. Next, additional node features from the VDJ-dataframe are added to the AntibodyForests-object with the function Af_add_node_feature. The influence of these parameters on the clustering is then analyzed with the function Af_cluster_node_features with the parameters: features = c("vgene_family", "timepoint", "isotype"), fill = "max", clusters = output$clustering (output from Af_compare_within_repertoires), and significance = T. These parameters take the most abundant node feature per tree in each cluster and calculates if there is a significant difference in cluster composition. Lastly, to analyze the potential difference in average distance to the germline of certain node features, we use the function Af_distance_boxplot with the parameters: distance = "edge.length", min.nodes = 10, node.feature = "timepoint" or “tissue”, groups = c("d28", "d60", "d110", "d201") or NA (when node.feature is “tissue”), significance = T, and unconnected = T. These create boxplot for the average sum of edge lengths per group per tree of at least 10 nodes and calculate the T-test p-value.

For **Figure S6**, we run the function Af_build() on all samples with the following parameters: sequence.columns = "VDJ_sequence_aa_trimmed", germline.columns = "VDJ_germline_aa_trimmed", and construction.method = "phylo.network.default". Next, we run the PLM Ablang (10) to construct probability matrices and calculate pseudolikelihoods using the code in this repository: https://github.com/dvginneken/PLM-pipeline. We use the function Af_add_node_feature to add the pseudolikelihoods to the AntibodyForests-object. The correlation between pseudolikelihood and distance to the germline was analyzed with the function Af_distance_scatterplot with the parameters: min.nodes = 5, color.by = “sample”, color.by.numberic = F, correlation = “pearson”. This creates a scatterplot of the pseudolikelihood of sequences in the trees with a least 5 nodes, the dots are categorically colored on the donor ID and a pearson correlation coefficient is calculated. Mutations along the edges of the tree were analyzed by supplying the PLM probability matrices to the function Af_PLM_dataframe and plotted with Af_plot_PLM with the parameters: group_by = “sample_id” and values = “substitution_rank”. The average ranks of the mutating and conserved residues were analyzed with the function Af_plot_PLM_mut_vs_cons with the parameters: values = "rank", dots =

"all_edges", group_by = "sample_id". Finally, the PLM likelihood patterns between samples were compared with the function Af_compare_PLM.

For Figure **S3**, the preprocessed BCR reads one of the mice from Neumeier et al. (9) were processed with the VDJ_build function of the Platypus package (version 3.6.0) in R (v4.4.0) (7). We applied the following parameters to the VDJ_build() function: trim.germlines = T, remove.divergent.cells = T, complete.cells.only = T. A data frame with the bulk transcript was integrated into this VDJ-dataframe with the AntibodyForests function VDJ_integrate_bulk using the parameters: organism = “mouse”, trim.FR1 = T, and tie.resolvement = “all”. This function annotates the single-cell and bulk transcripts using IgBLAST (12), trims the FR1 regions from all the sequences and reconstructed germline sequences to account for variation in primer design, and merges the bulk transcripts into the existing single-cell clonotypes based on identical CDR3 length, V and J gene usage, and CDR3 sequence similarity. We run Af_build on the integrated VDJ-dataframe with the parameters: sequence.columns = "VDJ_sequence_aa_trimmed", germline.columns = "VDJ_germline_aa_trimmed", construction.method = "phylo.network.default", parallel = F, node.features = c("dataset"). Next, we plot clonotype 3 and 5 using the function Af_plot_tree.

For Figure **S7**, we used the data from the largest binding IgG clone of Mouse1 from Neumeier et al. (9). A lineage tree was created in R (v4.4.0) with AntibodyForests Af_build using the parameters: sequence.column = c("VDJ_sequence_aa", "VJ_sequence_aa"), germline.columns = c("VDJ_germline_aa", "VJ_germline_aa"), node.features = c("octet.affinity"). Next, we predicted the antibody-antigen complex 3D structure of the heavy and light chain of each node together with the antigen Ovalbumin (SERPINB14) using AlphaFold3 (11). The resulting structures were superimposed on the C-alpha carbon positions. 3Di sequences were determined using mini3di (v0.2.1) (13) and pKa values and free energy were computed using propka (v3.5.1) (14), both in Python (v3.11.10). Structural properties were then calculated incorporated into AntibodyForests with the function VDJ_3d_properties using the parameters: properties = c("charge", "3di_germline", "hydrophobicity", "RMSD_germline", "pKa_shift", "free_energy", "pLDDT"), chain = "HC+LC" (for S7C left and S7E,F) or chain = "whole.complex” (for S7B,C right), sequence.region = "full.sequence" (for S7C left, S7D,E,F) or “binding.residues” (for S7C right.). These structural properties were added to the AntibodyForests object using the function Af_add_node_feature and distance to the germline was plotted with Af_distance_scatterplot with parameters: correlation = "pearson", color.by = "octet.affinity", color.by.numeric = T, geom_smooth.method = "lm". Figure S7F was created with the function Af_edge_RMSD.

Data visualization: Figures **1, S1, S4, S6A** and **S7A** were created with Biorender.com. Lineage trees in Figures **S2, S3, S6B**, and **S7B** were generated with the function Af_plot_tree. The heatmap of the across tree construction method in Figure **S4** was generated with Af_compare_methods. The heatmap in Figure **S5B** was generated with Af_compare_within_repertoires. The boxplots in Figure **S5C** were generated with Af_cluster_metrics. The barplots in Figure **S6C** were generated with Af_cluster_node_features. The boxplots in Figure **S5E** were generated with Af_distance_boxplot. The scatterplot in Figure **S6C, S7C,D,E** were generated with Af_distance_scatterplot. The distribution plots in Figures **S6D** was generated with Af_plot_PLM. The boxplot in Figure **S6E** was generated with

Af_plot_PLM_mut_vs_cons. The heatmap in Figure **S6F** was generated with Af_compare_PLM. The scatterplot in Figure **S7F** was generated with Af_edge_RMSD.

Data availability: Data for Figures **S2, S5** and **S6** was downloaded from the NCBI Sequence Read Archive using SRA Toolkit with ID PRJNA777934 (6). Data for Figures **S3** and **S7** was provided by Neumeier et al. (9)**.**

1. Hoehn KB, Pybus OG, Kleinstein SH. Phylogenetic analysis of migration, differentiation, and class switching in B cells. PLOS Computational Biology. 2022 Apr 25;18(4):e1009885.

2. Barak M, Zuckerman NS, Edelman H, Unger R, Mehr R. IgTree: creating Immunoglobulin variable region gene lineage trees. J Immunol Methods. 2008 Sep 30;338(1-2):67–74.

3. Schramm CA, Sheng Z, Zhang Z, Mascola JR, Kwong PD, Shapiro L. SONAR: A High-Throughput Pipeline for Inferring Antibody Ontogenies from Longitudinal Sequencing of B Cell Transcripts. Front Immunol. 2016 Sep 21;7:372.

4. DeWitt WS, Mesin L, Victora GD, Minin VN, Matsen FA. Using Genotype Abundance to Improve Phylogenetic Inference. Mol Biol Evol. 2018 Feb 20;35(5):1253–65.

5. Lindeman I, Emerton G, Mamanova L, Snir O, Polanski K, Qiao SW, et al. BraCeR: B-cell-receptor reconstruction and clonality inference from single-cell RNA-seq. Nature Methods. 2018 Jul 31;15(8):563–5.

6. Kim W, Zhou JQ, Horvath SC, Schmitz AJ, Sturtz AJ, Lei T, et al. Germinal centre-driven maturation of B cell response to mRNA vaccination. Nature. 2022 Feb 15;604(7904):141–5.

7. Cotet TS, Agrafiotis A, Kreiner V, Kuhn R, Shlesinger D, Manero-Carranza M, et al. ePlatypus: an ecosystem for computational analysis of immunogenomics data. Bioinformatics [Internet]. 2023 Sep 2;39(9). Available from: http://dx.doi.org/10.1093/bioinformatics/btad553

8. Hoehn KB, Vander Heiden JA, Zhou JQ, Lunter G, Pybus OG, Kleinstein SH. Repertoire-wide phylogenetic models of B cell molecular evolution reveal evolutionary signatures of aging and vaccination. Proc Natl Acad Sci U S A. 2019 Nov 5;116(45):22664–72.

9. Neumeier D, Yermanos A, Agrafiotis A, Csepregi L, Chowdhury T, Ehling RA, et al. Phenotypic determinism and stochasticity in antibody repertoires of clonally expanded plasma cells. Proc Natl Acad Sci U S A. 2022 May 3;119(18):e2113766119.

10. Olsen TH, Moal IH, Deane CM. AbLang: an antibody language model for completing antibody sequences. Bioinform Adv. 2022 Jun 17;2(1):vbac046.

11. Abramson J, Adler J, Dunger J, Evans R, Green T, Pritzel A, et al. Accurate structure prediction of biomolecular interactions with AlphaFold 3. Nature. 2024 May 8;630(8016):493–500.

12. Ye J, Ma N, Madden TL, Ostell JM. IgBLAST: an immunoglobulin variable domain sequence analysis tool. Nucleic Acids Res. 2013 Jul;41(Web Server issue):W34–40.

13. van Kempen M, Kim SS, Tumescheit C, Mirdita M, Lee J, Gilchrist CLM, et al. Fast and

accurate protein structure search with Foldseek. Nature Biotechnology. 2023 May 8;42(2):243–6.

14. Olsson MHM, Søndergaard CR, Rostkowski M, Jensen JH. PROPKA3: Consistent Treatment of Internal and Surface Residues in Empirical pKa Predictions. J Chem Theory Comput. 2011 Feb 8;7(2):525–37.
